# Modelling of stress distribution and fracture in dental occlusal fissures

**DOI:** 10.1038/s41598-019-41304-z

**Published:** 2019-03-18

**Authors:** Boyang Wan, Mahdi Shahmoradi, Zhongpu Zhang, Yo Shibata, Babak Sarrafpour, Michael Swain, Qing Li

**Affiliations:** 1 School of Aerospace, Mechanical and Mechatronic Engineering, The University of Sydney, Sydney, NSW 2006 Australia; 2 School of Computing, Engineering and Mathematics, Western Sydney University, Penrith, NSW 2751 Australia; 3 Department of Conservative Dentistry, Division of Biomaterials and Engineering, Showa University School of Dentistry, 1-5-8 Hatanodai, Shinagawa-ku, Tokyo 142-8555 Japan; 4 The University of Sydney, Discipline of Oral Surgery, Medicine and Diagnostics, School of Dentistry, Faculty of Medicine and Health, The University of Sydney, Westmead Centre for Oral Health, Westmead Hospital, Sydney, NSW 2145 Australia

## Abstract

The aim of this study was to investigate the fracture behaviour of fissural dental enamel under simulated occlusal load in relation to various interacting factors including fissure morphology, cuspal angle and the underlying material properties of enamel. Extended finite element method (XFEM) was adopted here to analyse the fracture load and crack length in tooth models with different cusp angles (ranging from 50° to 70° in 2.5° intervals), fissural morphologies (namely U shape, V shape, IK shape, I shape and Inverted-Y shape) and enamel material properties (constant versus graded). The analysis results showed that fissures with larger curved morphology, such as U shape and IK shape, exhibit higher resistance to fracture under simulated occlusal load irrespective of cusp angle and enamel properties. Increased cusp angle (i.e. lower cusp steepness), also significantly enhanced the fracture resistance of fissural enamel, particularly for the IK and Inverted-Y shape fissures. Overall, the outcomes of this study explain how the interplay of compositional and structural features of enamel in the fissural area contribute to the resistance of the human tooth against masticatory forces. These findings may provide significant indicators for clinicians and technicians in designing/fabricating extra-coronal dental restorations and correcting the cuspal inclinations and contacts during clinical occlusal adjustment.

## Introduction

Dental enamel, as the most highly mineralised tissue in the human body, mainly consists of hydroxyapatite nanocrystals and traces of water and organic molecules. The crystalline structure as well as the high hardness and stiffness of enamel impart it with the capacity to tolerate a range of lifelong mechanical, thermal and chemical stimuli while still maintaining its cutting and masticatory functions. However, due to its highly mineralised composition, enamel is a relatively brittle biomaterial with an elastic modulus in the level of 100 GPa and a toughness value comparable to that of glass^1^. The brittle nature and high susceptibility to mechanical failure presents a more critical problem in deep fissures located in the occlusal surface of molar and premolar teeth. In these fissural regions, the thickness of enamel is significantly lower than enamel cusp tips and proximal surfaces. Moreover, the sharp angles and narrow curvatures within the fissure system potentially generate vulnerable sites of stress concentration and crack initiation in enamel.

The cusps of posterior teeth bear substantial bending and flexural loads during functional and specifically eccentric parafunctional occlusal movements. These applied loads can theoretically generate a horizontal force component which tends to open up the fissural space and create tensile stress concentration and subsequent cracks at the base of the fissure. Although it is common to see that cracking of the cusps initiates from the fissural area in carious teeth, the prevalence of fracture in the fissural area of sound natural teeth is surprisingly low^2^. The resistance of fissural enamel with presumably weak structural design and brittle nature, against millions of chewing cycle loads with up to 1200 N in magnitude is a thought-provoking phenomenon.

While occlusal enamel fissures seem to be susceptible sites for bacterial plaque retention, caries formation and crack initiation, their introduction into the dentition of heterodonts was a significant step in evolution which led to the transformation of canine-like teeth morphology of zyphodonts to the multicuspid dentition of heterodonts, with a broad diversity of tooth types and shapes^3^. This transformation enhanced the efficacy of the masticatory system of omnivores and optimised the utilisation of food caloric energy for an increased level of intricate physical and intellectual activities^4^. Natural remineralisation process through salivary minerals and organic biomolecules is a reparative mechanism for maintaining the integrity of enamel and restoring mildly demineralised crystals^5^.

The genesis of occlusal fissures happens during the bell stage of the tooth formation process, through folding invagination of the inner enamel epithelium and the subsequent fusion and mineralisation of adjacent developmental dental lobes^6^. While these fissures can display a range of depths and widths, from shallow grooves and fossae to deep and narrow pits and fissures, their morphology follows five main patterns^7^, namely I-shape, U-shape, inverted Y-shape (IY), IK-shape and V-shape.

Previous studies have revealed that enamel thickness and cusp size are the critical morphological factors involved in the fracture behaviour of enamel^8^. Other studies have focused on the microstructural features of enamel including its hierarchical organisation, the presence of micron-sized defects and the role of organic remnants, in the mechanical resistance and fracture behaviour of enamel^8,9^. However, little has been known to date about the effect of fissure geometry as well as the composition and material properties of enamel especially around the fissural area, on the resistance of the tooth against fracture. Understanding the structural and compositional features responsible for the superior mechanical performance of natural teeth, can provide substantial guidelines for optimizing design and fabrication of long lasting restorative ceramic crowns. Therefore, the current study aims to investigate the fracture behaviour of fissural dental enamel under simulated occlusal load in relation to various factors, including fissure morphology, cuspal angle and the underlying material properties of enamel.

## Results

The influence of anatomical features and compositional factors, such as fissure types, cusp angulation and material properties on the stress distribution, fracture load and crack length in the occlusal fissure of the tooth, are presented in the following sections.

### Fissure geometry

**Figure 1:**
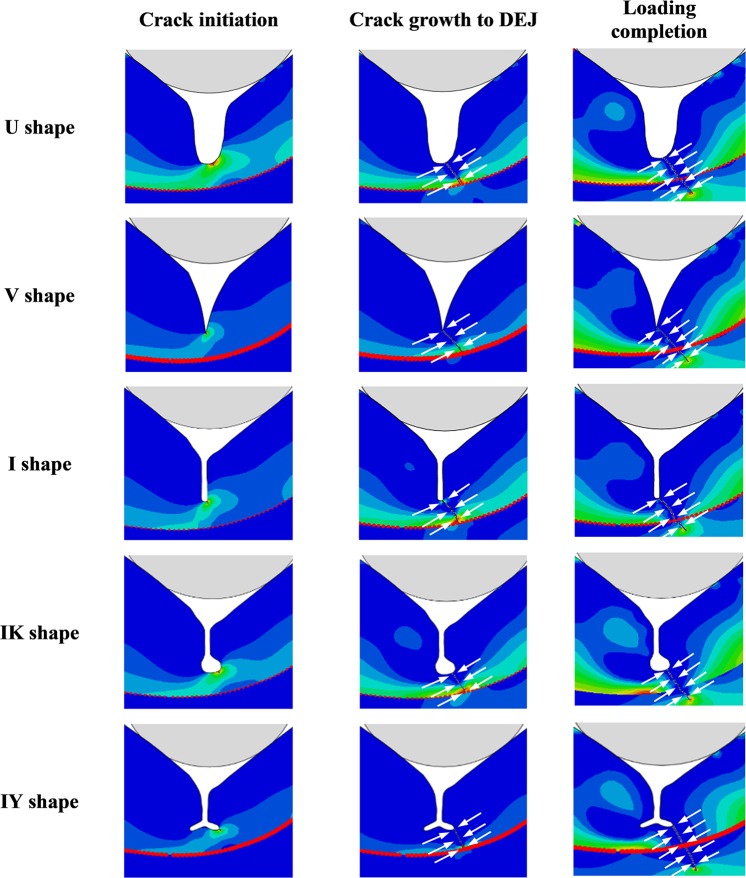
Patterns of crack propagation for tooth models and the relevant maximum principal stress contour at different stages of crack formation including crack initiation, crack growth to DEJ and crack extension upon completion of loading. From top to bottom, the models have the same cusp angle (50°), but different fissure geometries, namely U, V, I, IK and IY shapes.

The contours of the maximum principal stress associated with crack initiation and propagation for the tooth models with the five types of fissure morphology at a cusp angle of 50° are shown in Fig. 1. The crack patterns at different stages of crack development, including crack initiation, crack growth to dentine-enamel junction (DEJ), and crack extension upon completion of indenter displacement loading were recorded for these five fissure geometries. For all the fissure shapes, the crack generally started from the floor of the fissure where high tensile stresses occurred. Mostly, it was observed that the cracks initiated on one side of the fissure, and then propagated in the same direction. The cracks stopped growing once they reached the DEJ, which is marked as the red line in each sub-figure of Fig. 1. With continuation of the loading, the crack passed through the DEJ and continued to grow in dentine. Consequently, the crack stopped in the dentine region when the displacement of the loading ball (0.2 mm) and the resultant loading was completed.

**Figure 2:**
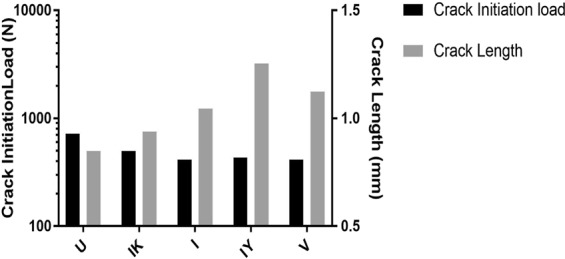
Crack initiation load (left Y axis) and crack length at completion of loading displacement (0.2 mm) (right Y axis) for five fissure geometries, including U, IK, I, IY and V shape.

In the XFEM analysis, the reaction force on the spherical loading head was recorded as the fracture load^10^. As such, the loads corresponding to onset of the fissure crack are graphed in Fig. 2. On the basis of the fracture loads at crack initiation, the U shape fissures showed the highest value, followed by the IK shape. The remaining three geometries (V shape, I shape and Inverted Y shape) presented lower but similar loads. Regarding the crack length, as shown by the grey bars in Fig. 2, the longest extension was 1.25 mm which corresponds to the IY shape, followed by the V shape (1.12 mm), I shape (1.04 mm), IK shape (0.94 mm) and U shape (0.84 mm).

### Cusp angle

**Figure 3:**
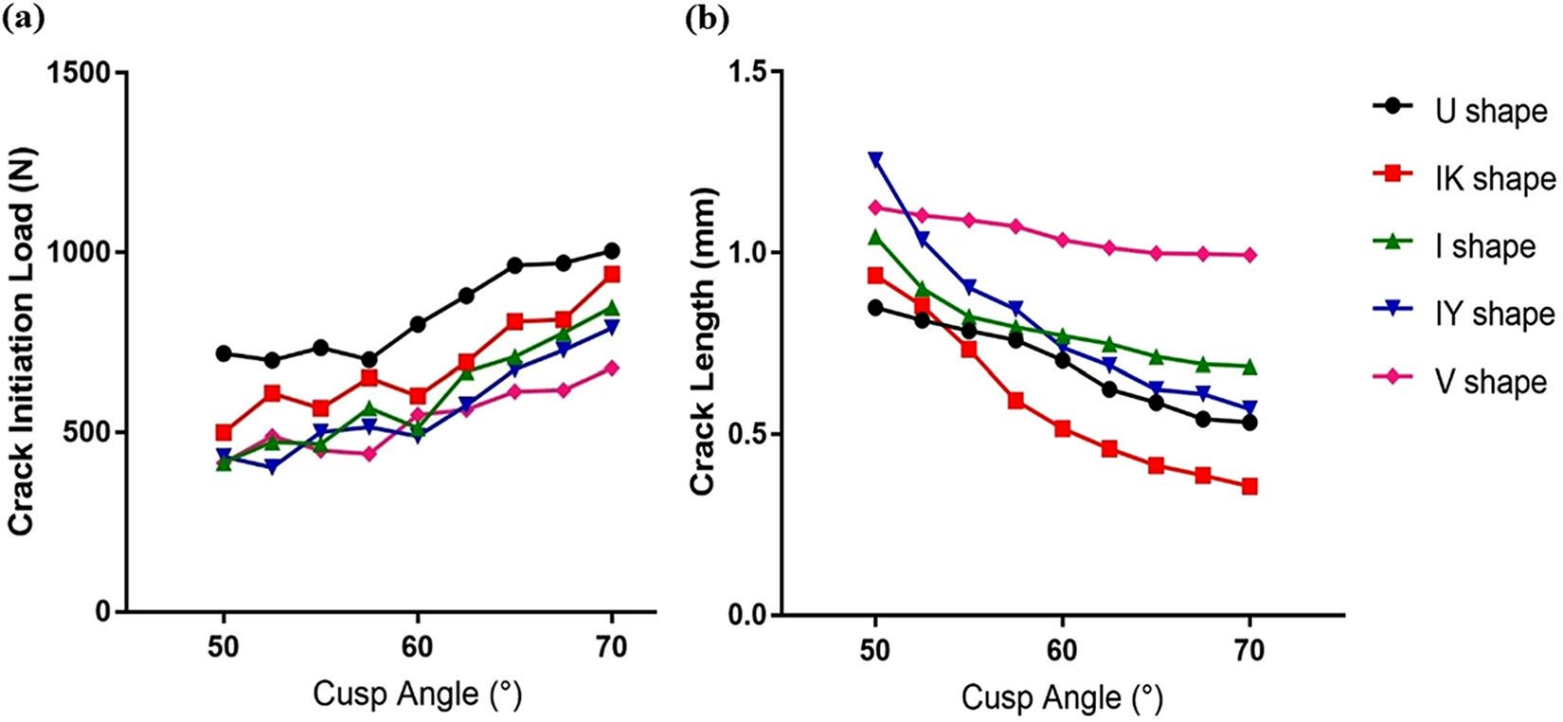
(**a**) Comparison of the fracture load at crack initiation; (**b**) Crack length at completion of loading, for the models with different fissure geometries and different cusp angles (from 50° to 70° at interval of 2.5°).

The fracture loads at the onset of crack initiation were recorded from the XFEM analysis for different cusp angles (ranging from 50° to 70° at intervals of 2.5°) of each fissure geometry, as graphed in Fig. 3(a). It was observed that the fracture load rises with the increase in cusp angle (i.e. lower cusp steepness) for all fissure shapes. For the entire range of cusp angles, the U shape morphology resulted in the highest fracture load at crack initiation, followed by the IK shape. For the remaining three fissure types (V shape, I shape and IY shape), the fracture loads at crack initiation were similar when cusp angle was at the low value of 50° (Fig. 3(a)). Upon increasing the cusp angle up to 70°, the load values at crack initiation were somewhat scattered for these three geometries with a decreasing rank of I to IY and V shape fissures.

By comparing the fracture initiation loads between the cusp angles of 50° and 70°, a relatively large difference was observed for the IK shape, I shape and IY shape (Fig. 3(a)). For all the fissure geometries, the crack initiation load was relatively stable for the cusp angles between 50° and 57.5°; and then increased for the cusp angles from 57.5° to 70°. Regarding the IK shape, the loads at crack initiation were fluctuating and increasing. For the I shape and IY shape, the loads fluctuated when the cusp angle was between 50° and 60°, and increased when the cusp angle was between 60° and 70°.

Crack lengths and maximum load upon completion of the loading displacement were also recorded from the XFEM analysis for the different fissure geometries and various cusp angles (Fig. 3(b)). Comparative results of the crack length among all five fissure shapes are shown in Fig. 2 for the cusp angle of 50°. Figure 3(b) graphs the crack length values upon completion of loading for different cusp angles (from 50° to 70°). Accordingly, the crack lengths decreased with the increase in cusp angle for all the fissure geometries. The V shape had the least change in the crack length for different cusp angles between 50° and 70°. The slope of decreasing trend of the U shape was greater than that of the V shape, which was fairly stable within the entire cusp angle range. The IY shape, I shape, and IK shape had a similar trend, i.e. crack lengths declined sharply when cusp angle changed from 50° to 57.5°, followed by a smaller rate of crack length reduction in the cusp angle range of 60° to 70°. The reduction of the crack length was more significant in the IK and IY shape fissures, where the crack length almost halved when increasing the cusp angle from 50° to 70°. At the low cusp angle of 50°, the crack length of V shape fissures was significantly larger than those of the other fissure types which exhibited a decreasing rank of I shape, IY shape, U shape and IK shape.

### Material properties of enamel

The fracture behaviour of enamel in relation to the gradient of mineral density and corresponding material properties of enamel in different parts of the tooth such as the fissure area were investigated for different fissure geometries. Accordingly, the fracture loads at different stages of crack formation were recorded for the models with constant material properties throughout the enamel in comparison with the models having an allocated CT-based gradient of material properties in enamel.

**Figure 4:**
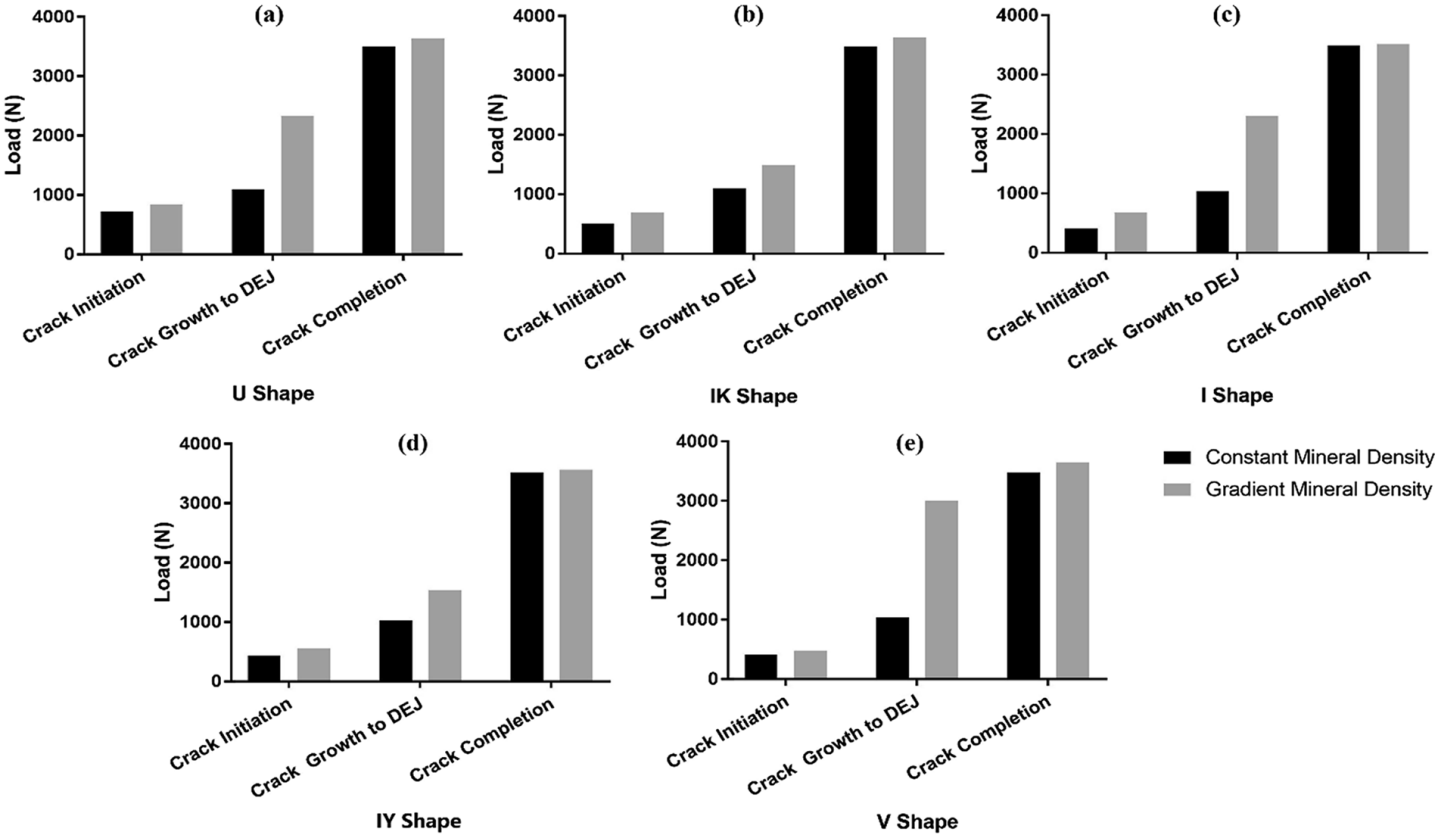
Fracture load at crack initiation, crack growth to DEJ and crack completion stages for the models with constant and graded mineral density and material properties at a cusp angle of 50°.

Figure 4 shows the comparative results of the fracture loads at crack initiation, crack growth to DEJ and upon completion of loading displacement for these models with the constant and graded material properties at a cusp angle of 50°. In general, the models with a gradient of mineral density and graded material properties showed a trend of increased fracture loads in all three stages of crack formation in comparison with the similar models with constant mineral density. The effect of graded versus constant mineral density was more significant in the second stage of crack development (crack propagation to DEJ) compared with the crack initiation and maximum crack extension at the displacement completion stages. In the crack initiation and loading completion stages, none of the fissure geometries were overly sensitive to the changes in the mineral density, where both the constant and gradient density models had relatively similar fracture loads. However, in the stage of crack growth to DEJ, the application of gradient density on the models significantly increased the load values for the U shape, V shape and I shape, to almost twice that of the models with constant density (Fig. 4). The graded mineral density also resulted in an increase of the loads for crack growth to the DEJ in the IK shape and IY shape models, however the effect was relatively small compared to the other three geometries.

**Figure 5:**
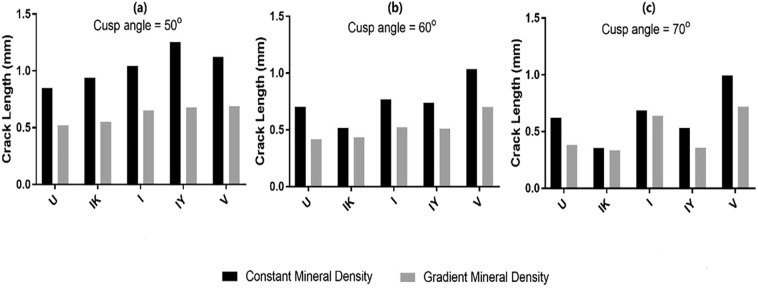
Crack length of the models with constant and graded mineral density for the different fissure types at cusp angles of 50°, 60° and 70°.

Further to investigate the combined effect of fissure geometry, cuspal angle and material properties of enamel on the fracture behaviour of occlusal fissures, the crack length at the completion of the loading displacement was recorded. Figure 5 shows the comparative results of the crack length for the different fissure types at cusp angles of 50°, 60° and 70° for the models with constant and graded mineral density, respectively. It was noted that in general, the crack length in the models with graded mineral density was shorter than that in the constant density models regardless of cusp angle and fissure geometry.

At a low cusp angle of 50°, application of a CT-based gradient of mineral density had significant effect on the fracture resistance and led to a sizeable decrease in the crack length for all the fissure geometries. For both the gradient and constant mineral density patterns at 50° angle, the U shape geometry had the smallest crack length, followed by the IK shape, I shape and V shape. The IY shape demonstrated the most substantial crack length of all the fissure geometries, indicating lowest fracture resistance.

While the IY shape was the most affected geometry by the gradient mineral density at the low cusp angle of 50°, mineral density gradient did not have a significant influence on the crack length for the I shape and IK shape fissures at a cusp angle of 70°. In these fissure types, the effect of graded mineral density became smaller with increasing cusp angle from 50° to 70°. The influence of gradient density on the crack extension was evident in all cusp angles for the U shape and V shape fissures.

At the high cusp angle of 70°, gradient mineral density led to smaller crack lengths for the U shape, V shape and IY shape. As for the IK shape and I shape, change in mineral density had limited effect on the crack length.

## Discussion

Cracking and fracture of the tooth imposes a significant concern in the dental clinic, with a questionable prognosis for tooth fracture in the majority of patients^11^. This issue is more frequently diagnosed in teeth with deteriorated structural integrity due to caries or large intra-coronal restorations^12^. However, the rate of fracture is very low in sound teeth despite the presence of deep fissures on the occlusal surface as well as thin brittle enamel in these areas of the tooth. This study investigated the interaction of various anatomical features and compositional factors which contribute to the resistance of the fissural enamel against fracture.

As shown in Fig. 1, in all the study groups, the cracks initiated at the fissure area where the maximum tensile stress was concentrated; and then propagated toward the right lower direction in the enamel, presumably due to the non-symmetrical structure of the modelled tooth. The non-symmetrical anatomy of the models were created to reflect the structural asymmetry of the natural premolar tooth in which the functional cusp is generally smaller and has a rounder shape in comparison with the sharper and larger non-functional cusp. The directional propagation of the crack toward the smaller cusp can be attributed to smaller bulk of the enamel and dentine together with the lower thickness of enamel, which leads to higher stress concentration.

Regarding the effect of occlusal fissure geometry on fracture resistance of the tooth, the results of the XFEM analysis showed that the fissures with larger curved morphology such as U and IK shape, exhibited higher resistance to fracture under simulated occlusal load irrespective of cusp angle and enamel material properties. At the crack initiation stage, these two fissure types required higher fracture loads to generate the crack. This may be explained by their curved morphology that distributed the stress over a wider area and prevented stress concentration around localised points in the fissure. On the other hand, the I, IY and V shape fissures displayed similarly lower fracture loads and longer crack lengths at the maximum load displacement due to their sharp and pointed shape at the tip of the fissure which prevented wider distribution of the stress and generated greater stress concentration.

The cusp angle is an important anatomical feature of the crown morphology which can be modified and adjusted in natural tooth and restorative crowns. Previous studies have evaluated the relation of cusp angle with the fracture resistance of ceramic crowns and the values between 50° and 70° have been suggested^13^ to be an optimum range of cusp angle. However, to the knowledge of the authors, the effect of cusp angles in combination with the fissure morphology and gradient properties of natural teeth have not been investigated so far.

As shown in Fig. 3(a), increasing the cusp angle results in an increase of the fracture resistance of the tooth. This finding is consistent with the mechanical test conducted on all ceramic porcelain veneered crowns with zirconia core by Sornsuwan *et al*.^14^. The applied occlusal force can be resolved into two components in the lateral and longitudinal (axial) directions. While higher cusp angulation is more related to the compressive stress and longitudinal (vertical) forces, steeper cusp inclination generates larger lateral (horizontal) forces in the fissure area which is the dominant factor in crack initiation and propagation. In the current study, the crack initiation load was almost unchanged for the cusp angles between 50° and 58°, but became sensitive to the cusp angle above 58° (Fig. 3(a)). The authors assume that at the cusp angle values below 58°, the tensile stress caused by the lateral (horizontal) force component is large enough to initiate and propagate the crack for all the fissure geometries. However, the crack length at the maximum displacement was still proportionate to the cusp angle value even in this low range of cusp angle values (Fig. 3(b)).

Material properties play an important role in crack growth of structures. While some studies have considered enamel as a homogenous material^15^, others have suggested enamel to be a functionally graded material (FGM) with a gradient of material properties^16,17^. In the present study, non-destructive microCT imaging was used to obtain mineral density maps in various sections of the tooth and subsequently generated Young’s modulus and fracture toughness maps of the specimen. Young’s modulus is a critical material property which influences the stress and strain energy, thereby determining crack initiation and propagation.

The XFEM procedure consisted of two stages; the first was crack initiation which simulated the crack nucleation and small crack growth; and the second stage modelled the propagation of the crack. Regarding crack initiation, materials with a lower modulus would allow more elastic deformation, i.e. more compliant (typically less brittle)^18^. Lower Young’s modulus leads to a lower tensile stress in the material; and therefore it would be more difficult for the crack to initiate. For the crack propagation, the determinant factor is the strain energy release rate (G)^19^, which is inversely proportional to Young’s modulus and directly proportional to the fracture toughness squared (Eq. 2). According to the mineral density maps of the tooth, enamel in the fissural region has a relatively lower Young’s modulus and higher fracture toughness than the outer enamel area. As a result, the crack would be more difficult to initiate and it would propagate more slowly under loading displacement in the fissural area.

As shown in Fig. 4, graded material properties had a more significant impact on the fracture load during the crack growth stage compared to the crack initiation and maximum crack extension stages. This observation is consistent with the known graded properties of enamel. The lower E modulus and higher K_1c_ and strain energy release rate results in a crack requiring more load to grow across the enamel layer before reaching the DEJ. Regarding the load at crack completion, nevertheless, there was no significant difference in the fracture loads. This is due to the fact that the crack had already passed the DEJ and entered the dentine for both the constant property and graded enamel property groups and therefore the crack opening displacement is controlled by the strain energy release rate of the dentine. The reduction of fracture load due to the graded material properties was more manifest for a lower cusp angle, particularly in the fissures with V and I shape, where the increase of the cusp angle is not able to impart significant resistance against fracture.

As shown in Fig. 5, if the cusp angle is in the lower range of the suggested values, graded material properties could be one of the ways to increase the resistance of the tooth to crack growth. However, with the increase in cusp angle, the effect of material properties on crack length became smaller due to the lower tensile stress.

According to these findings, application of materials with higher fracture toughness around the occlusal grooves can be an effective strategy to lower the risk of fracture for the intra-coronal restoration or crowns under heavy masticatory loads when the cusp angle cannot be increased. While in theory, restorative crown designs with curved occlusal fissure morphology such as the U and IK shapes provide higher fracture resistance and better stress distribution, however in practice it is very difficult, if not impossible for the dentists and technicians, to develop such fine features on the occlusal surface of the ceramic crowns. Though, avoiding the creation of sharp fissures and narrow morphological features on the occlusal surface of the restoration is of utmost importance to the enhancement of durability and resistance of the tooth and restoration.

Based upon the findings of this study, the authors suggest three mechanisms have been incorporated into the natural design of the tooth, imparting it with the maximum resistance against masticatory loads while still maintaining the benefits of a multicuspid dentition with narrow and deep occlusal fissures.

The first mechanism is the rounding and radial extension of the fissure tip which allows more even distribution of the stresses over a larger area. The second mechanism involves the increase of the cusp angle (reduction of the cusp steepness) which has developed/evolved more noticeably in highly load bearing molar teeth compared to premolars. Interestingly, the gradual wear and attrition of the occlusal surfaces in subjects with high masticatory load level, which leads to the increase of the cusp angles, can be considered as a protective mechanism that can reduce the risk of cracking and tooth fracture in these subjects.

Finally, the gradient of mineral density and material properties within enamel, i.e. higher toughness of inner and fissural enamel, provides a way to offset the destructive consequences of stress concentration caused by the sharp morphology around fissure tips. The collective effect of these anatomical and compositional features along with the intricate microstructural properties such as hierarchy, remnant proteins, orientation and bundling of hydroxyapatite crystals makes enamel an extraordinary material with many unique properties.

## Materials and Methods

### Mineral density characterization and quantification

Extracted human premolar teeth were collected from oral surgery and orthodontic departments at Sydney Dental Hospital, The University of Sydney. The specimen collection and experimental methods were performed in accordance with the approval guidelines from Sydney local health district ethics review committee, protocol No X12–0065 & HREC/12/RPAH/106. Regarding specimen collection, each patient was provided an information sheet and informed consent was obtained from all subjects.

Mineral density distributions of teeth were characterised using a high-resolution desktop microscopic computed tomography system (Skyscan 1172, Skyscan N.V, Aartselaar, Belgium) at an accelerating source voltage of 100 keV, a source current of 100 μA, and an exposure time of 885 ms. Three hydroxyapatite discs with low, medium and high mineral densities were used for grey level calibration to determine the mineral density values of different parts of the tooth. Details of the employed phantoms are provided elsewhere^20^.

Low energy X-rays were eliminated using an inbuilt filter equal to 1.0 mm thickness of aluminium and 0.05 mm of copper to restrict the spectral bandwidth of the polychromatic radiation. The equivalent monochromatic energy spectrum of filtered X-rays had an effective mean energy of 60 keV. The long axes of the teeth were placed parallel with the centre of rotation of the mounting device. During the scanning process, the samples were rotated over 360° at angular increments of 0.14°, generating 2570 two-dimensional shadow projections with an image matrix of 2000 pixels × 1048 pixels. These images were saved as 16-bit Tagged Image File Format (TIFF) and consequently exported to a 3D cone beam reconstruction program (NRecon software, version 1.4.4; SkyScan) for the tomographic reconstruction of the tooth and production of tomographic images. The tomographic reconstruction produced a dataset of slice views in 16-bit TIFF format, which were perpendicular to the specimen rotation axis and had a voxel size resolution of 8–10 µm. Vertical bucco-lingual slice views were produced by re-slicing the reconstructed volume of the whole image stack in FIJI (W.S. Rasband, U.S. National Institutes of Health, Bethesda, Md, USA, http://imagej.nih.gov/ij/, 1997–2011).

The produced tomographic images were consequently imported into MATLAB (MatLab R2012b 8.0.0.783, Mathworks, Natick, MA, USA) for the de-noising process which increases the signal to noise ratio of the micro-CT images. De-noising was performed using a method based upon total variation regularisation^21,22^, with the following parameters: (regularisation parameter) = 0/04.  (initial penalty parameter) = 3.

The calibration of mineral density was implemented by measuring and averaging grey level values of selected images of each hydroxyapatite phantom, followed by plotting the obtained grey level values against the mineral density value of that phantom. Based upon the plotted values, a calibration equation was used to transform the grey level values of the images into true mineral density values^20^.

Mineral mapping and colour-coding of the tooth was performed using the Colormap editor command in MATLAB by choosing Jet colour map with fixed RGB (Red, Green, Blue) index values for all of the colourized images. The colour codes were based upon the greyscale values and corresponding mineral densities of the specimen, thereby yielding a calibrated mineral map.

### Finite element models

**Figure 6:**
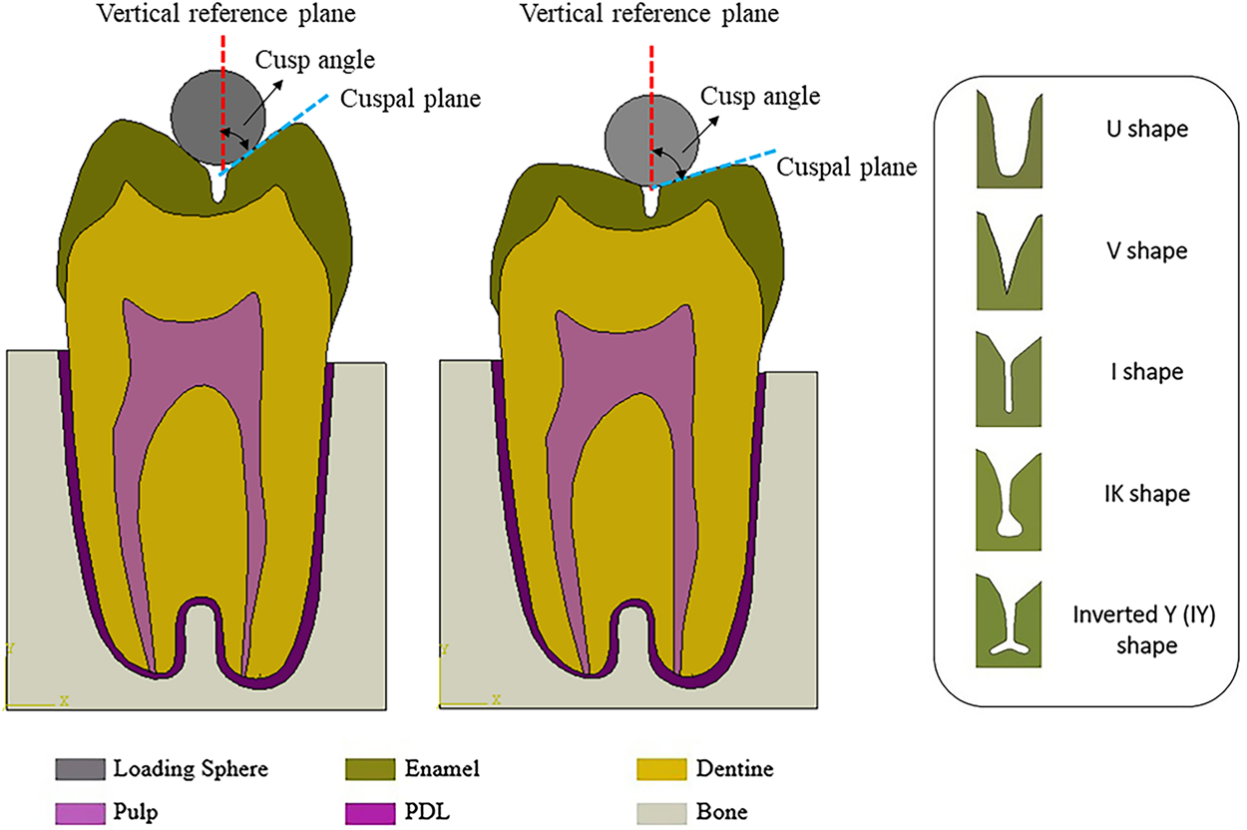
Finite element models of a premolar tooth with the U-shape occlusal fissure: (**a**) cusp angle 50°. The cusp angle is defined as the angle between the slope of the cusp (blue dotted line) and the vertical reference plane (red dotted line); (**b**) cusp angle 70°; (**c**) Five geometry types of occlusal fissure. The colours in the figure represent the bone, loading sphere and different parts of the tooth.

A bucco-lingual cross-sectional image was selected from the micro-CT dataset of an upper premolar tooth to construct the two-dimensional (2D) finite element (FE) models^23^. The anatomies of bone, enamel and dentine, were specified in the image processing software ScanIP (Simpleware Pty. Ltd, UK). Once surface refinement of masks for each component of the model was completed, the FE model was exported as an IGES file for further modification in SolidWorks (SolidWorks Corp., USA). The periodontal ligament (PDL) was then created by offsetting the outline of the root section of the tooth^13,24^. Five different geometries for the occlusal fissure including U shape, V shape, IK shape, I shape and Inverted Y (IY) shape were modelled as shown in Fig. 2(c). For each fissure geometry, nine FE models with varying cusp angles from 50° to 70° at intervals of 2.5° were constructed in SolidWorks. Nominated models of a tooth with the U shaped occlusal fissure and cusp angles of 50° and 70° are shown in Fig. 6(a and b), respectively.

The FE models were meshed by using four-node bilinear quadrilateral elements. The mesh convergence test was conducted to determine the appropriate mesh size for tooth models by balancing the accuracy of results and computational cost^24,25^. According to the results of the convergence test, the global mesh size was 0.09 mm for the tooth model and 0.2 mm for the loading sphere. Due to the complex geometry of fissure areas, the meshing refinement was further performed using a local mesh size of 0.03 mm. Consequently, the number of elements was 42,282 (Degree of freedom - DOF: 84,736) for the tooth model, and 440 (DOF: 934) for the loading sphere.

The models were loaded using a 5 mm diameter steel ball controlled by a downward displacement of 0.2 mm. As this finite element analysis was carried out to simulate the indentation process, the sphere model played the role of indenter ball. Therefore, the sphere model was set to be the a rigid body and the contact between sphere (indenter ball) and tooth surface was assigned a friction coefficient of 0.3 in tangential direction^11^. All the FE models were kinematically constrained on the outer and bottom surfaces of the bone region^26,27^.

### Material properties of the components of the FE model

**Figure 7:**
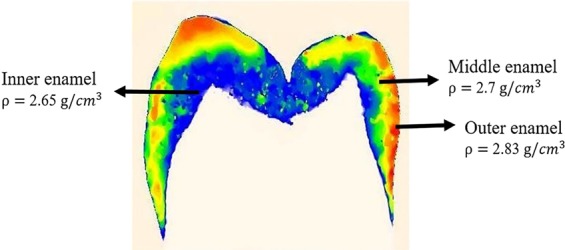
Colourized mineral map (contour) of a buccolingual section of a premolar tooth, generated from a micoCT image. The mineral map (contour) displays the gradient of mineral density decreasing from enamel surface towards the dentine-enamel junction.

The loading sphere was treated as steel, which was modelled to be linear elastic, isotropic and homogeneous^28^. Regarding the tooth model, all the materials were considered to be isotropic and homogeneous^11^. However, for the enamel, three regions of inner, middle and outer enamel were considered separately in order to reflect the non-isotropic nature as well as the gradient of the mineral density^6^ and material properties which exist in natural enamel. These areas were determined based upon the mineral maps generated from microCT images of the tooth. Figure 7 shows the nominated colorized mineral map of a buccolingual section of a premolar tooth. The mineral map (contour) displays the gradient of mineral density decreasing from enamel surface towards the dentine-enamel junction.

Table 1 shows the values for the mineral density and corresponding Young’s modulus, fracture toughness and strain energy release rate in different areas of enamel.

**Table 1:** Material properties of the components in the FE model are adopted from the literature^3,6,41,42^.

Material	Young’s Modulus *E* (MPa)	Poisson’s Ratio *v*	Tensile Stress *σ*_*TS*_ (MPa)	Fracture Toughness *K*_*IC*_ (MPa·m^1/2^)	Strain energy release rate *G* (J/m^2^)	Mineral Density ρ (g/*cm*^3^)
Steel	200000	0.3	—	—	—	—
Dentine	19800	0.31	70	3.15	110	—
Pulp	10	0.49	—	—	—	—
PDL	70.3	0.45	—	—	—	—
Bone	12200	0.26	—	—	—	—
Uniform Enamel	84,100	0.3	50	1.05	12.7	2.83
Outer enamel	84,100	0.3	50	0.67	4.86	2.83
Middle enamel	70,000	0.3	50	1.6	36.26	2.7
Inner enamel	60,000	0.3	50	2.5	94.80	2.65

Previous studies have quantified the relationship between mineral density and Young’s modulus in enamel^3,6,29^. Accordingly, the mineral density of enamel lies mainly within the linear portion of the mineral density versus elastic modulus curve^18^; and therefore, elastic modulus values can be calculated from the values of calibrated mineral density. The previous studies have also shown that the fracture toughness of enamel is correlated with the distribution of mineral density^30^. However, in contrast to the trend of Young’s modulus, the higher density values translate to lower fracture toughness values^30,31^.

The critical strain energy release rate *G* (J/m^2^) is a material parameter related to the fracture toughness *K*_*IC*_ (MPa·m^½^), indicating the energy required to propagate unit area of a crack. For a 2D plane-strain model, strain energy release rate (*G*) can be calculated as follows:

1 $$G=\frac{{K}_{IC}^{2}}{{E}^{\ast }}$$

2 $${E}^{\ast }=\frac{E}{1-{\nu }^{2}}$$

where *E* is the Young’s modulus, ν is Poisson’s ratio and *K*_*IC*_ is the fracture toughness of the material.

### Extended finite element method (XFEM)

Fracture analysis signifies an important field in engineering and has gained growing popularity in dental materials and biomechanics research recently. Ichim *et al*.^32^ modelled crack initiation and propagation for a range of case scenarios, such as contact-induced damage in layered materials systems and the failure of enamel, dentine, restored tooth and all-ceramic dental bridges. Barani *et al*.^33,34^ investigated longitudinal cracking in enamel by XFEM, in which the simulated cracks were in agreement with the experimental observations of the crack growth in human teeth. Li *et al*.^35^ compared the CDEM and XFEM for studying the fracture behaviours of all-ceramic dental prostheses, showing the cons and pros of each method.

In this present study, the XFEM fracture analysis was performed to evaluate the formation of cracks in the occlusal fissure area using Abaqus 6.14 (ABAQUS, Inc, Providence, RI). The cracking criterion used here was the maximum principal stress criterion as^36^,

3 $${f}^{e}=\frac{{\sigma }_{1}^{e}}{({\sigma }_{max}^{0})}$$

where ${\sigma }_{max}^{0}$ represents the allowable maximum principal stress of the materials (enamel and dentine), ${\sigma }_{1}^{e}$ is the maximum principal stress in element (*e*) and ${f}^{e}$ indicates the stress ratio, determining if cracking would initiate in the element. A crack was assumed to occur when the maximum principal stress exceeds the prescribed tensile strength of the material. The subsequent crack growth was based upon the strain energy release rate^37^. The cracking region was considered in both enamel and dentine in the XFEM models. Detailed background information about the mechanics of XFEM fracture analysis can be obtained from the literature^38–40^.
